# The *de Novo* Reference Genome and Transcriptome Assemblies of the Wild Tomato Species *Solanum chilense* Highlights Birth and Death of NLR Genes Between Tomato Species

**DOI:** 10.1534/g3.119.400529

**Published:** 2019-10-11

**Authors:** Remco Stam, Tetyana Nosenko, Anja C. Hörger, Wolfgang Stephan, Michael Seidel, José M. M. Kuhn, Georg Haberer, Aurelien Tellier

**Affiliations:** * Phytopathology, Technical University Munich, Germany,; † Population Genetics, Technical University Munich, Germany,; ‡ Plant Genome and Systems Biology, Helmholtz Center of Munich, Germany,; § Environmental Simulations, Helmholtz Center of Munich, Germany,; ** Department of Biosciences, University of Salzburg, Austria,; †† Evolutionary Biology, LMU Munich and Natural History Museum Berlin, Germany, and; ‡‡ Evolutionary Biology and Ecology, Albert-Ludwig University of Freiburg, Germany

**Keywords:** Genome sequence assembly, Transcriptome, Evolutionary Genomics, Tomato, NLR genes

## Abstract

Wild tomato species, like *Solanum chilense*, are important germplasm resources for enhanced biotic and abiotic stress resistance in tomato breeding. *S. chilense* also serves as a model to study adaptation of plants to drought and the evolution of seed banks. The absence of a well-annotated reference genome in this compulsory outcrossing, very diverse species limits in-depth studies on the genes involved.

We generated ∼134 Gb of DNA and 157 Gb of RNA sequence data for *S chilense*, which yielded a draft genome with an estimated length of 914 Mb, encoding 25,885 high-confidence predicted gene models, which show homology to known protein-coding genes of other tomato species. Approximately 71% of these gene models are supported by RNA-seq data derived from leaf tissue samples. Benchmarking with Universal Single-Copy Orthologs (BUSCO) analysis of predicted gene models retrieved 93.3% of BUSCO genes. To further verify the genome annotation completeness and accuracy, we manually inspected the NLR resistance gene family and assessed its assembly quality. We find subfamilies of NLRs unique to *S. chilense*. Synteny analysis suggests significant degree of the gene order conservation between the *S. chilense*, *S. lycopersicum* and *S. pennellii genomes*.

We generated the first genome and transcriptome sequence assemblies for the wild tomato species *Solanum chilense* and demonstrated their value in comparative genomics analyses. These data are an important resource for studies on adaptation to biotic and abiotic stress in *Solanaceae*, on evolution of self-incompatibility and for tomato breeding.

Tomato (*Solanum lycopersicum*) is an important vegetable crop. Together with its wild relatives it is a model sytstem for tolerance to abiotic and biotic stresses (Tomato-Genome-Consortium 2012; Lin *et al.* 2014). Tomato breeders have often used germplasm of wild relatives to enhance stress tolerance (Bolger *et al.* 2014b). Genome assemblies exist for tomato relatives *S. habrochaites*, *S. pimpinellifolium* and *S. pennellii*. Yet, fully accessible and annotated reference genome sequences are to date only available for *S. lycopersicum* (Tomato-Genome-Consortium 2012) and the selfing wild tomato relative *S. pennellii* (Bolger *et al.* 2014b) Here, we present a reference genome assembly, annotation and additional *de novo* leaf transcriptome assemblies for the stress tolerant and outcrossing species, *S. chilense*.

*S. chilense* occurs on the southern edge of the wild tomato species range, in southern Peru and northern Chile. It belongs to the section Peruvianum, which contains four closely related wild tomato species, of which *S. chilense* forms a monophyletic subclade (Pease *et al.* 2016). *S. chilense* split from *S. peruvianum* about 1 mya (Arunyawat *et al.* 2007; Städler *et al.* 2008). Since then, the species has migrated southward and colonized diverse arid habitats both in mountainous and coastal terrain bordering the Atacama desert characterized by low temperatures or extreme aridity, respectively (Böndel *et al.* 2015)(Figure 1).

**Figure 1:**
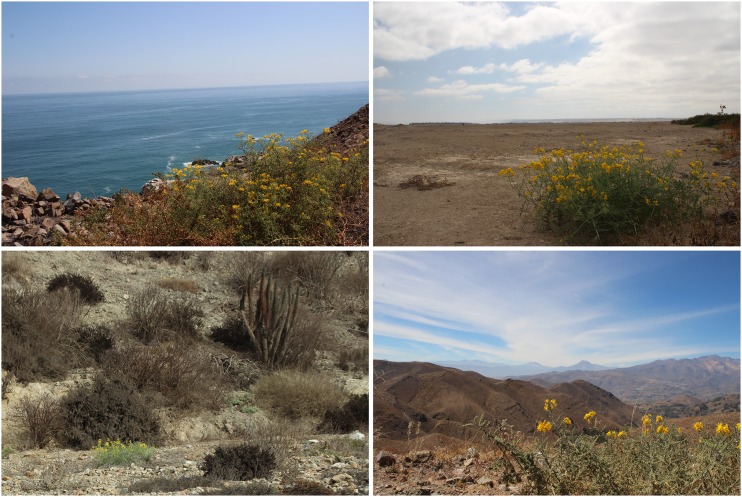
*Solanum chilense* populations in their natural habitat (by R. Stam). The top panels show coastal and lowland habitats; the lower panels show typical mountain habitats. LA3111 originates from a mountainous habitat similar to the last panel.

*S. chilense* has been used in studies on drought (Xia *et al.* 2010), salt (Zhou *et al.* 2011; Martínez *et al.* 2012) and cold tolerance (Nosenko *et al.* 2016), pathogen resistance and resistance gene evolution (Stam *et al.* 2017, 2019), as well as adaptation to extreme environments (Fischer *et al.* 2013; Böndel *et al.* 2018). As an outcrossing species it has been used to understand self-incompatibility in the tomato clade (Igic *et al.* 2007). The species is characterized by high levels of genetic diversity (Arunyawat *et al.* 2007; Städler *et al.* 2008; Böndel *et al.* 2015) probably due to existence of seed banking (Tellier *et al.* 2011).

*S. chilense* is also being used as a resource in tomato breeding; genes from *S. chilense* have been successfully introgressed to enhance resistance to the fungal pathogen *Verticilium dahliae* (Tabaeizadeh *et al.* 1999) and to the Tomato Mosaic Virus Y (Verlaan *et al.* 2013).

To corroborate the quality of our reference genome and to demonstrate its value for future genomic studies, we compared the NLR family in *S. chilense* with those in *S. lycopersicum* and *S. pennellii*. Canonical pathogen resistance genes in plants belong to the NLR family (Nod-like receptor or Nucleotide binding site, leucine rich repeat containing receptor) (Jones *et al.* 2016). NLRs are modular and contain an N-terminal domain, which can be a Toll-Interleukin Receptor (TIR) or a Coiled Coil (CC) domain, followed by a Nucleotide Binding Site (NBS) domain and several Leucine Rich Repeats (LRR). Complete NLRs have all three domains, whereas partial NLRs lack one or the other. Complete as well as some partial NLRs are involved in signaling of the plant immune system (Baggs *et al.* 2017). TIR- and CC-domain-containing NLRs are called TNL or CNL, respectively; CNL are often grouped in subclasses.

Comparative studies in *S. lycopersicum* and some wild relatives revealed interspecific differences in NLRs (Andolfo *et al.* 2014). For example, *S. lycopersicum* and closely related *S. pimpinellifolium*, contain respectively 326 and 355 NLRs, while *S. pennellii* contains only 216 putative NLRs (Stam *et al.* 2016). These differences in the NLR repertoire are thought to be the result of a birth and death process (Michelmore and Meyers 1998) and could possibly be explained by differences in pathogen pressure.

## Methods and Materials

### De novo genome sequence assembly for S. chilense LA3111

Seeds were obtained through the tomato genome resource center (TGRC, UC Davis). DNA was extracted from leaves of one adult plant from accession LA3111 (plant number 3111_t13) using the Qiagen DNAeasy kit following the instructions of the supplier. The sequencing was conducted at Eurofins Genomics (Ebersberg, Germany) using standard library preparation protocols for four different libraries. Two standard paired-end libraries were produced with insert sizes of 300 bp and 500-550 bp and two libraries were prepared for mate pair sequencing, with insert sizes of 8 kb and 20 kb. The 500 bp fragment library was sequenced using a MiSeq protocol, and overlapping paired-end reads (∼55%) were stitched to longer single reads using the software PEAR v0.9.8 (Zhang *et al.* 2014). Remaining unstitched clusters (45%) were retained as paired-end reads. The other three libraries were sequenced on Illumina HiSeq2500 sequencers at Eurofins Genomics. Construction of the mate-pair-like library was done at Eurofins Genomics using their proprietary protocol. Other libraries were constructed using commercially available kits (NEBNext Ultra DNA Library Prep Kit for Illumina, article number E7370) according to the manufacturer’s instructions. In brief, 1 µg of DNA was fragmented using a Covaris Instrument (Covaris Inc., Woburn, MA) according to manufacturer’s instructions. End-repair, A-tailing and ligation of indexed Illumina Adapter, size selection and amplification was performed accordingly. The resulting fragments were cleaned up and quantified. Libraries were loaded on the cBot (Illumina, San Diego, CA) and cluster generation was performed using manufacturer’s instructions. Sequencing of paired-end reads of 125 bp read length was performed on a HiSeq2500 machine (HiSeq Control Software 2.2.38) using HiSeq Flow Cell v4 and TruSeq SBS Kit v4. Raw data were processed using RTA v.1.18.61. CASAVA v.1.8.4 was used to generate FASTQ-files.

In total we generated ∼134 Gb of raw data (Table S1), which would correspond to > 130x coverage assuming a ∼950 Mb genome size, as was estimated by Eurofins Genomics using k-mer analysis using their proprietary protocol. In addition we used GenomeScope (Vurture *et al.* 2017) on our paired-end illumina HiSeq read data (recommended settings, k-mer size 27). We used the Celera assembler (CAv8.3; https://sourceforge.net/projects/wgs-assembler/files/wgs-assembler/wgs-8.3) and stitched and unassembled MiSeq read data to generate contigs. The fragment correction module and the bogart unitigger of the Celera assembler were applied with a graph and merge error rate of 5%. Minimal overlap length, overlap and merge error rates were set to 50 bp and 6% each, respectively. The resulting contigs were linked to scaffolds by SSPACE v2 (Boetzer *et al.* 2011) using all four libraries available for la3111_t13. Scaffolds were further processed by five iterations of GapFiller v2.1.1 and corrected by Pilon v1.21 in full-correction mode (Boetzer and Pirovano 2012; Walker *et al.* 2014).

Scaffolds containing exclusively organellar genes were detected using BLAST similarity searches (ncbi-blast v2.6.0+; e-value 1e-30) (Altschul *et al.* 1997) against the database containing chloroplast- and mitochondria-encoded genes of three *Solanaceae species*: *S. lycopersicum*, *S. pennellii* and *N. tabacum*.

### De novo assembly of S. chilense leaf transcriptome

Twenty four Illumina paired-end read RNA-Seq libraries were generated for 12 *S. chilense* plants from populations LA3111 and LA2750. Replicates were obtained by propagating plants vegetatively. Total RNA was extracted from leaf tissue samples from multiple mature plants under normal and stress (chilling, 6h at 4°) conditions using the RNeasy Plant Mini Kit (Qiagen GmbH, Hilden, Germany) and purified from DNA using the TURBO DNA-free Kit (Ambion, Darmstadt, Germany). RNA concentration and integrity were assessed using a Bioanalyzer 2100 (Agilent Technologies, Waldbroon, Germany). The preparation of random primed cDNA libraries has been performed by GATC Biotech AG (Konstanz, Germany) according to their internal and proprietary SOP. Sequencing on a HiSeq 2500 in paired end mode with a read length of 2 × 100 bases was also conducted by GATC Biotech AG.

RNA-Seq library contamination with non-target species and organellar RNA was assessed using FastQ Screen v0.5.2 (http://www.bioinformatics.babraham.ac.uk/projects/fastq_screen/) with 100000 read sub-sample and a database consisting of UniVec database and reference genome sequences of *Homo sapiens*, *Escherichia coli*, *Nicotiana tabacum* (NCBI) and *Fusarium graminearum* (ftp://ftpmips.helmholtz-muenchen.de/fungi/Fusarium/F_graminearum_PH1_v32). Quality of all RNA-Seq libraries were assessed using FastQC v0.11.2 (Andrews 2010). Adapters, reads with average quality below 30 and low quality (< 30) bases at the read termini were trimmed using Trimmomatic v0.35 (Bolger *et al.* 2014a). Only paired reads with minimum after-processing length of 70 bp were retained and used for assembling transcriptomes. Data for each population (six individuals and 12 RNA_seq libraries per population) were assembled *de novo* using Trinity v2.3.2 (Grabherr *et al.* 2011), SOAPdenovo-Trans v1.0.4 (Xie *et al.* 2014) and Oases-Velvet (v0.2.08/v1.2.08) (Schulz *et al.* 2012); the redundancy acquired from pooling the three assemblies was reduced using the EvidentialGene pipeline (Gilbert 2013); SOAPdenovo-Trans assembly was conducted using k-mer sizes from 29 to 79 with a step size of 10. Oases-Velvet assembler was run with k-mer sizes from 25 to 73 with a step size of 12. Trinity assembly was conducted using the k-mer size of 25, the default and the only size supported by this assembler. Oases and SOAP assemblies obtained with each k-mer size were pulled together and clustered using CD-HIT-EST v4.6.5 and identity parameter set at 100% (Li and Godzik 2006). The contigs output by CD-HIT-EST and contigs resulting form the Trinity assembly were combined together and clustered using the EvidentialGene pipeline. Because FastQ Screen analyses identified organellar (chloroplast and mitochondrial) RNA as the major source of the RNA-Seq library contamination (up to 15%; Figure S1), contigs resulting from the assembly of organellar sequences were identified using BLAST similarity searches (ncbi-blast v2.6.0+; e-value 1e-70) against the chloroplast- and mitochondria-encoded proteins of three *Solanaceae species*: *S. lycopersicum*, *S. pennellii* and *N. tabacum*, and were excluded from the resulting *de novo* transcriptome assemblies.

### Gene model prediction

We applied a previously described consensus approach (Wang *et al.* 2014) to derive gene structures from the *S. chilense* draft genome. Briefly, *de novo* gene finders Augustus v3.2.3 (Stanke *et al.* 2006), Snap v1.0beta (Korf 2004) and GeneID v1.4.4 (Parra *et al.* 2000) were trained on a set of high confidence models that were derived from the LA3111 and LA2750 transcriptome assemblies. Existing matrices for eudicots and *S. lycopersicum* were used for predictions with Fgenesh v1.6 (Salamov and Solovyev 2000) and GlimmerHMM v3.0.1 (Majoros *et al.* 2004), respectively. Predictions were weighted by a decision tree using the JIGSAW software v3.2.10 (Allen and Salzberg 2005). Spliced alignments of known proteins and *S. chilense* transcripts of this study were generated by the GenomeThreader tool v1.6.6 (Gremme *et al.* 2005). We used current proteome releases (status of August 2016) of *Arabidopsis thaliana*, *Medicago truncatula*, *Ricinus communis*, *S. lycopersicum*, *Glycine max*, *Nicotiana benthiamiana*, *Cucumis sativa* and *Vitis vinifera*. Spliced alignments required a minimum alignment coverage of 50% and a maximum intron size of 50 kb under the *Arabidopsis* splice site model. Next, *de novo* and homology predictions were merged to top-scoring consensus models by their matches to a custom reference blastp database comprising *Arabidopsis*, *Medicago*, *S. lycopersicum* and *S. pennellii* proteins. In a last step, we annotated the top-scoring models using the AHRD (“A human readable description”)-pipeline (Wang *et al.* 2014) and InterProScan v5.21 (Zdobnov and Apweiler 2001) to identify and remove gene models containing transposon signatures. The resulting final models were then classified into high scoring models if they showed at least 90% of alignment consistency for both query (*S. chilense*) and subject (custom plant database gene as described above), an evalue of 1e-30 or better and at least 60% of the bitscore of the blastp comparison.

To obtain an additional RNA-seq support for the predicted gene models, raw RNA-seq data were processed (adapter and quality trimming) using Trimmomatic v0.35 (Bolger *et al.* 2014a) and aligned to the *S. chilense* genome sequence assembly using STAR v2.5 (Dobin *et al.* 2013). Read pairs aligned to exonic regions of predicted gene models were summarized per gene using featureCounts (Liao *et al.* 2014). To account for differences in library sizes, the resulting count matrix was normalized using DeSeq2 (Love *et al.* 2014). Genes represented by at least ten normalized counts (*i.e.*, read pairs) in at least three samples were considered expressed.

Functional gene annotation and assignment to the GO term categories were performed using Blast2GO v 4.1 (Conesa and Götz 2008) based on the results of InterProScan v5.21 (Zdobnov and Apweiler 2001) and BLAST (ncbi-blast v2.6.0+) (Altschul *et al.* 1997) similarity searches against the NCBI non-redundant sequence database. KEGG pathway orthology assignment of protein-coding genes was conducted using KAAS (Moriya *et al.* 2007).

The completeness of the *S. chilense* genome and transcriptome sequence assemblies and annotation was assessed using Benchmarking with Universal Single-Copy Orthologs (BUSCO v3) analysis (Simão *et al.* 2015). As an additional criterion for assessing genome assembly and annotation, we evaluated the composition and quality of the NLR gene model prediction in *S. chilense*.

Loci encoding putative NLR genes were identified using NLRParser (Steuernagel *et al.* 2015) with cut-off thresholds as described before (Stam *et al.* 2016). We manually inspected all regions with NLR motifs and updated the annotated open reading frames where this was required. The improved annotation was based on presence of NLR motifs and expression evidence (from the RNA-seq data). For example, in some cases the automated annotations contained introns that led to exclusion of NLR domains or missed adjacent annotated NLR domains, whereas our RNA-Seq confirmed that these domains were transcribed. In these cases we either removed annotated introns or selected alternative predicted ORFs to contain the largest number of NLR domains, without introducing frame shifts. Only 15 NLR genes required manual curation based on the RNA-Seq data aligned to the reference genome. In ten instances, frame shifts made it impossible to enhance the gene model. For these genes, the computationally predicted CDS were retained. The remaining 211 predicted NLR gene models showed to be well resolved and did not require any correction. Functional clades were assigned based on protein sequences of the NBS domain. BLASTp searches (cutoff e-value 10^−30^) were used to match the *S. chilense* NLRs with the nearest previously assigned NLRs (Andolfo *et al.* 2014). For example, the clade of the best hit in other tomato species was assigned to the *S. chilense* gene. In the majority of cases, this gave unequivocal results. In one instance, members of our new clade matched two previously defined clades equally well; this clade thus has double naming (CNL1/CNL9). The NLRs in two identified clusters did not match any NLRs that had been clustered previously. In these cases, new cluster numbers were assigned (CNL20, CNL21).

To assess synteny between the *S. chilense*, *S. lycopersicum* and *S. pennellii* genomes, orthologous pairs of protein-coding genes were identified using reciprocal BLAST searches with an e-value threshold of 10^−30^ and maximum target sequence number 50. For *S. lycopersicum* and *S. pennellii*, the longest splice variant for each gene was used as a BLAST input. Duplicated BLAST matches were filtered out. Only gene pairs confirmed by bidirectional BLAST searches were retained and used as an input for synteny analyses. A spatial distribution of resulting orthologous gene pairs was analyzed and gene blocks conserved between genomes (syntenic) were identified using iADHoRe v3.0.0.1 (hybrid mode with minimum syntenic block size = 3; (Proost *et al.* 2012)). For tandem arrays of genes, a single representative was retained in syntenic blocks. To compare, we also conducted synteny analyses using DAGchainer (r02-06-2008; Haas *et al.* 2004) with the following parameters: a constant match score of value 12 (-Z 12), gap penalty equal to 1 (-g 1), minimum chain length equal five colinear genes (-A 5) and allowing neighboring genes within a single chain to be no more than ten genes apart (-D 10).

### Phylogenetic analyses

**Figure 2:**
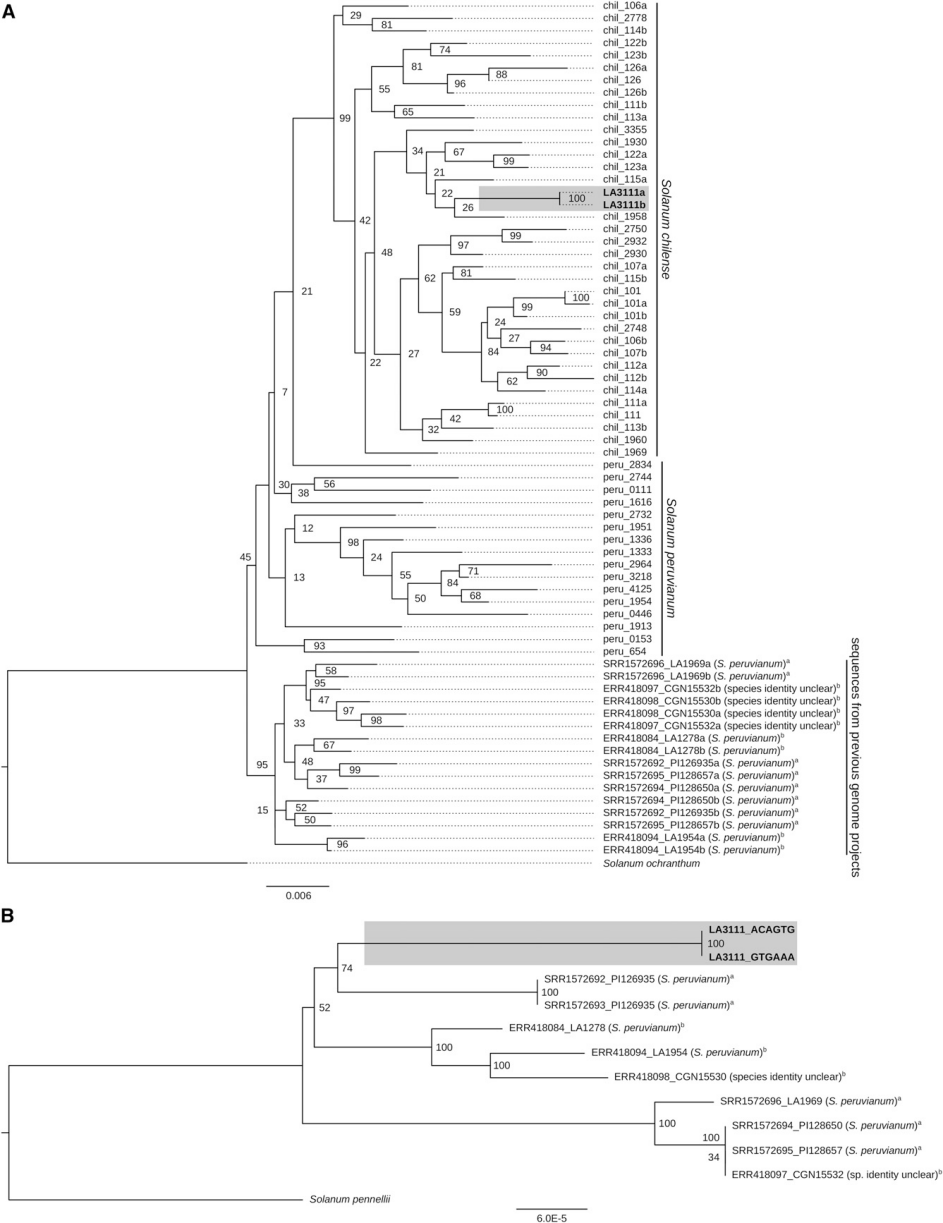
A) Maximum Likelihood (ML) phylogenetic tree constructed based on six CT loci (nuclear genes) extracted from the *S. chilense* LA3111 sample sequenced in this study (in bold; marked with gray rectangle) and previously sequenced *S. peruvianum* and *S. chilense* samples. For the samples from whole genome data, sequences were aligned to the *S. pennellii* reference genome and the sequence data for the CT loci was extracted. Single-gene alignments were concatenated; the resulting super alignment was used as in input for RaxML to construct the ML tree (1000 bootstrap replicates). The branch length is shown as expected number of substitutions per site and bootstrap values are reported on each tree node. *S. ochranthum* was used as an outgroup. The sequence IDs containing *chil* and *peru* represent Sanger sequences from *S. chilense* and *S. peruvianum* individuals, respectively, followed by the accession/ individual number. The sequences with IDs containing SRR- and ERR-numbers followed by the accession number were extracted from previously generated whole genome data. B) Phylogeny constructed based on chloroplast SNP data extracted from *S. chilense* LA3111 (in bold and marked with gray rectangle) and previously sequenced *S. peruvianum* whole genome sequence data. Chloroplast sequences were aligned to the *S. pennellii* reference genome. The tree was built using resulting alignments and PhyML (GTR, NNI, BioNJ, 1000 bootstrap replicates). The branch length is shown as expected number of substitutions per site and bootstrap values are reported on each tree node. ^a^Sequences of individuals with IDs containing SRR1572692, SRR1572693, SRR1572694, SRR1572695 and SRR1572696 were obtained from Lin *et al.* (2014). ^b^Sequences with IDs containing ERR418084, ERR418094, ERR418097 and ERR418098 originate from 100 Tomato Genome Sequencing Consortium (2014). Individual SRR1572696 was reported as *S. chilense* in the main text of the paper (ref) and as *S. peruvianum* in the supplementary, which contain all original data. The original classification of the sequences with IDs ERR418097 and ERR418098 as *S. chilense* has been later withdrawn from the CGN database.

We mapped our sequence data as well as data from all nine publicly available *S. peruvianum* and presumed *S. chilense* data (100 Tomato Genome Sequencing Consortium 2014; Lin *et al.* 2014) (SRA numbers: ERR418084, ERR418094, ERR418097, ERR418098, SRR1572692, SRR1572692, SRR1572694, SRR1572695 and SRR1572696, origins described in Figure 2) against the *S. pennellii* reference genome (Bolger *et al.* 2014b) using STAMPY v1.0.2 (Lunter and Goodson 2011) (substitution rate 0.01, insert size 500). The SNP calling and filtering was done using samtools v0.1.19 (mpileup, call -m with default parameters) (Li *et al.* 2009).

We extracted two types of loci. First, we extracted the sequence at six CT loci (CT066, CT093, CT166, CT179, CT198, CT268) for all 12 accessions. These are single-copy cDNA markers developed and mapped in Tanksley *et al.* (1992), which have previously been used to investigate the evolutionary relationships between wild tomato species (*e.g.*, Arunyawat *et al.* 2007; Rose *et al.* 2011; Böndel *et al.* 2015). To account for heterozygosity, two alleles were constructed randomly per individual. A concatenated alignment was prepared and manually checked. We added to this alignment 53 sequences obtained by Sanger sequencing in previous work on *S. chilense* and *S. peruvianum* (Städler *et al.* 2008). These sequences originate from *S. chilense* or *S. peruvianum* accessions as identified by the TGRC (UC Davis, USA) according to the taxonomic key in Peralta *et al.* (Peralta *et al.* 2008). *S. ochranthum* (accession LA2682) was used as an outgroup. The phylogentic reconstruction (Figure 2A) was obtained by the Maximum Likelihood method (ML; with GTR+Gamma+I model and 1000 bootstrap replicates) as implemented in RaxML (Stamatakis 2014).

Second, we reconstructed the sequences for the coding regions of the chloroplast for each of the 12 samples listed above and our LA3111 sample. These sequences were aligned and a phylogenetic tree was constructed using PhyML (Guindon *et al.* 2010) (ML, GTR, 1000 bootstraps, Best of NNI&SPR, BioNJ). The resulting tree was visualized and edited using Figtree v1.4.4 (Rambaut and Drummond 2009).

### Data availability

The *S. chilense* genome data and raw RNA-Seq data generated for this study are deposited to the NCBI Short Read Archive under the BioProject IDs PRJNA508893 and PRJNA474106. All supplementary figures and tables presented in the manuscript, as well as the raw output files for BUSCO and KEGG, the NLR synteny and the .phy files used in the phylogenies are available on figshare. The *S. chilense* genome sequence assembly and annotation, CDS and protein models and *de novo* leaf transcriptome assemblies (for the accessions LA3111 and LA2750) are available via NCBI (assembly ASM601370v1) and through the Sol Genomics Network.

(ftp://ftp.solgenomics.net/genomes/Solanum_chilense/Stam_et_al_2019/). Supplemental material available at figshare: https://doi.org/10.25387/g3.8223794.

## Results and Discussion

### First S. chilense genome sequence assembly

Species within the Peruvianum group have diverged relatively recently and exhibit high intraspecific genetic and phenotypic diversity (Pease *et al.* 2016). Hence, species assignment of individuals from this complex can be ambiguous (Zuriaga *et al.* 2008). To confirm that our newly sequenced plant is indeed *S. chilense*, we performed phylogenetic comparisons of our sequenced individual and publicly available sequence data from *S. chilense* and *S. peruvianum*. We find that all robustly assigned *S. chilense* accessions (Städler *et al.* 2008) and our LA3111 individual cluster together into a well-supported monophyletic group (Figure 2A), while the recently sequenced accessions from 100 Tomato Genome Sequencing Consortium (2014) and Lin *et al.* (2014) form a polyphyletic group with known *S. peruvianum* samples.

Additionally, we reconstructed the chloroplast phylogeny of the members of the *S. peruvianum* clade. All previously sequenced samples formed a polyphyletic group, which is a topology known for the species *S. peruvianum*, whereas our *S. chilense* sample forms a separated branch (Figure 2B). Thus, phylogenetic analyses of both nuclear- and plastid-encoded genes confirm that data presented in this study are the first instance of a *S. chilense* genome sequence assembly.

### S. chilense LA3111 genome and transcriptome statistics

The final contig assembly comprised 150,750 contigs ranging from 1 to 162 kb totalling ∼717.7 Mb of assembled genome sequence with a N50 of 9,755 bp. After further processing, the 81,307 final scaffolds span a total size of 914 Mb with a N50 of 70.6 kb (Table 1). Of them, 111 scaffolds of total length ∼730 kb contain exclusively organellar genes and potentially represent the *S. chilense* chloroplast and mitochondrial genomes (Table S7). Our total genome size is very similar to the size calculation using k-mer analysis (∼950 Mb) and a bit larger than the estimates obtained by GenomeScope (Vurture *et al.* 2017) (721 Mb, Figure S2). The genomes for *S. lycopersicum* (Tomato-Genome-Consortium 2012) and *S. pennellii* (Bolger *et al.* 2014b) and are estimated to be ∼900 and ∼940 Mb, respectively. GenomeScope has been shown to underestimate the size for very heterozygous species like *S. chilense* (*e.g.*, for oyster, which is also highly heterozygous, flow cytometry, k-mer analyses and GenomeScope analyses esitmated 637, 545 and 471 Mb respectively (GenomeScope manual; Vurture *et al.* 2017). Therefore, we expect the genome size of *S. chilense* to be in the range of 900-950 Mb.

**Table 1:** S. chilense genome assembly

Total size (Mbp)	913.89
Scaffolds	81,307
N50 Scaffolds (bp)	70,632
Max Scaffold length (bp)	1,123,112
High confidence gene loci	25,885

The resulting transcriptome assemblies contain 41,666 and 35,470 transcripts for LA3111 and LA2750, respectively (Table 2). Despite the fact that both *S. chilense* transcriptome assemblies were derived from a single tissue and only two conditions (normal and cold-stress), they are 93.3% (LA3111) and 94.2% (LA2750) complete according to the BUSCO analyses (Table S2). Our data included RNA-Seq libraries for 24 *S. chilense* individuals. High variation of gene expression within- and between populations in the wild plant species might have contributed to the relative richness of the resulting transcriptome assemblies.

**Table 2:** S. chilense *de novo* transcriptome assemblies

	*S. chilense* transcriptome	
**Statistics**	**LA3111**	**LA2750**
Total contig number	41,666	35,470
Minimum length (bp)	123	123
Maximum length (bp)	16,476	16,473
Average length (bp)	831	943
Median length (bp)	504	684
N50 (bp)	1383	1458
N90 (bp)	351	432

We predicted 25,885 high-confidence (hc) gene loci that show good coverage and high homology to known proteins of other tomato species. Besides their support by homology, approximately 71% (18,290) of the hc genes are additionally supported by RNA-seq data derived from leaf tissue samples. Complementary to the set of hc models, we report the presence of 41,481 low confidence (lc) loci to maximize gene content information. Functionality for some of the lc models (6,569) is suggested by transcriptome evidence from the leaf RNA-seq data.

### Completeness and gene model validation

The completeness of the assembled genome was assessed using BUSCO (Simão *et al.* 2015) and was at 91.8%. This number is lower than the scores found for the previously annotated *S. lycopersicum* and *S. pennellii* genomes (Table S3), which is an expected difference between genome assemblies generated from the highly heterozygous individuals of self-incompatible species and inbred individuals of selfing species.

Synteny analyses using both iADHORE and DAGchainer showed conserved gene order between the genomes of the three tomato species: *S. chilense* (this study), *S. lycopersicum* (NCBI genome annotation release 102, ITAG2.4) and *S. pennellii* (NCBI genome annotation release 100, v2). We found that our *S*. *chilense* gene models (hc and lc) show homology to respectively 24,651 *S. lycopersicum* and 25,695 *S. pennellii* genes. By different estimates, from 2,364 to 3,216 (iADHoRe and DAGchainer, respectively) syntenic blocks containing total from 12,984 to 19,090 unique gene pairs were formed between the *S. chilense* and *S. pennellii* genomes (Table S4). Comparison with the *S. lycopersicum* genome output similar results: from 2,535 to 3,225 syntenic blocks containing from 14,013 to 19,114 gene pairs, respectively (Table S5). To compare, 977 syntenic gene blocks were detected between *S. lycopersicum* and *S. pennellii* genomes using the same i-ADHoRe parameters consisting of 18,064 and 17,904 gene models, respectively (Table S6). According to the results of the DAGchainer analysis, which has a higher block selection stringency relative to i-ADHoRe, syntenic blocks between *S. chilense* and both *S. pennellii* and *S. lycopersicum* contain at average 5.9 gene pairs; over 69% *S. chilense* scaffolds are represented by a single syntenic block containing at average 8.5 genes (maximum 136 genes; for both comparisons). Results of these analyses show preservation of positional association between genes among the three *Solanum* genomes. However, relatively high fragmentation of the *S. chilense* genome assembly does not allow us to assess large chromosomal re-arrangements in this species relative to *S. pennellii* and *S. lycopersicum*.

### NLR annotation and comparison

In total we found 236 putative NLRs, of which 139 are CNLs and 35 TNLs. Sixty two NLRs cannot be assigned to either class. Most CDS were supported by all three measures used for the annotation. The total number of NLRs identified in *S. chilense the S. chilense genome* is lower than in cultivated tomato (355) and more similar to *S. pennellii* (216), (Stam *et al.* 2016). The syntenic blocks identified between the *S. chilense* and the *S. lycopersicum* and *S. pennellii* genomes include 69 and 50 NLR genes, respectively, and show that NLRs are distributed across all 12 chromosomes (Supplementary Material). Except for several short tandems of identical or nearly identical gene copies, NLRs do not tend to form any positional clusters in tomato genomes. Only 30% of *S. chilense* NLRs belong to syntenic gene blocks (compared to *S. lycopersicum* and *S. pennellii*) showing the fast evolution and genomic organization of this gene family at the phylogenetic time scale (over millions of years).

**Figure 3:**
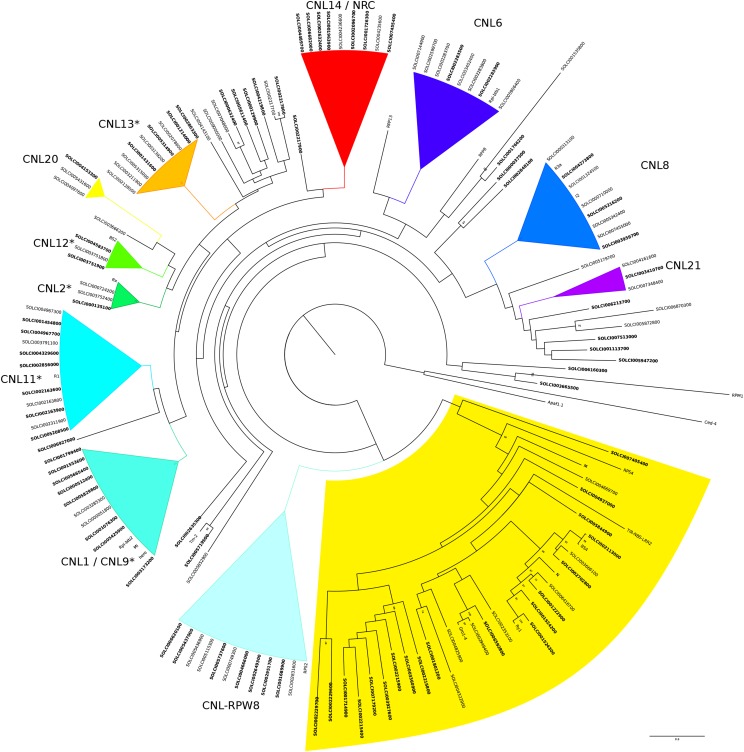
Maximum Likelihood (ML) phylogenetic tree of the NLR genes identified in *S. chilense*. The tree was made as described in Stam *et al.* 2016. Clades with high (>80%) bootstrap values are collapsed. Most previously described clades can be identified and are indicated as such. The TNL family is highlighted in yellow. Several previously identified NLR genes from different species are included for comparison and Apaf1.1 and Ced4 are used as an outgroup, similar as in Andolfo *et al.* (2014) and Stam *et al.* (2016). A list of these genes and their species of origin can be found in Table S8. Clades marked with an asterisk are NRC-dependent. NLR with orthologs (based on reciprocal best blast hits) in *S. pennellii* are in bold. The branch length is shown as expected number of substitutions per site. Clades CNL20 and CNL21 are new for *S. chilense*.

To further confirm the relative completeness of the NLR set in *S. chilense*, we reconstructed a phylogeny for the gene family based on the NBS protein sequences of the NLRs. All major NLR clades found in *S. lycopersium* and *S. pennellii* are present in the *S. chilense* genome (Figure 3). There are some small but interesting differences with the other tomato species. The CNL-4 and CNL-15 clades contained four or five members in *S. lycopersicum*, yet in *S. chilense* each had only one member. In addition, we identified two new clades: CNL20 and CNL21. A direct comparison shows that some clades have more members in *S. pennellii*, while others have more members in *S. chilense* (Figure S3). Similar differences can be seen between *S. pennellii* and *S. lycopersicum* (Stam *et al.* 2016) Using the definitions of Michelmore and Meyers (1998), our data suggest the birth and death of NLR genes between tomato species.

## Conclusions

We present the first draft genome sequence assembly and *de novo* transcriptome assemblies of the wild tomato species *S. chilense*. Using several complementary methods, including comparative analyses for a large and complex gene family such as the NLR-family, we show that quality of this genome assembly and annotation satisfy requirements for a reference genome for comparative genomics studies.
